# Effects of L-Arabinose on Glycemic Responses After the Consumption of Sucrose-Rich Foods in Individuals with Impaired Fasting Glucose: A Randomized Controlled Cross-Over Trial

**DOI:** 10.1016/j.tjnut.2025.06.028

**Published:** 2025-07-01

**Authors:** Leoné Pretorius, Korrie Pol, Corine Perenboom, Katherine M Appleton, Janet James, Monica Mars

**Affiliations:** 1 Department of Psychology, Bournemouth University, Bournemouth, United Kingdom; 2 Division of Human Nutrition and Health, Wageningen University, Wageningen, The Netherlands; 3 Department of Nursing Science, Bournemouth University, Bournemouth, United Kingdom

**Keywords:** L-arabinose, glucose, insulin, glucagon, continuous glucose monitoring, impaired fasting glucose

## Abstract

**Background:**

Impaired fasting glucose (IFG) is considered a preclinical stage of type 2 diabetes. L-arabinose is a sucrase inhibitor that interferes with sucrose breakdown and has been shown to lower glycemic and insulinemic responses in healthy individuals. However, its effects in individuals with IFG are unknown.

**Objectives:**

This study aims to assess effects of L-arabinose on glycemic responses after the consumption of sucrose-rich foods in individuals with IFG.

**Methods:**

Eighteen adults [4 females, 14 males; age 73 ± 4 y; body mass index 27.5 ± 2.4 kg/m^2^] with IFG participated in a double-blind, randomized, cross-over trial. Participants received 10% w/w L-arabinose (treatment) in a 550 mL sucrose drink or a sucrose-only drink (control). Blood glucose and insulin were measured before and ≤180 min post consumption. After this, participants consumed a 2-day controlled diet with sucrose-rich (9–10 en%) meals and snacks, preceded by a 15% w/w L-arabinose supplement or no L-arabinose (control). Continuous glucose monitoring (CGM) assessed glycemic variability throughout the trial.

**Results:**

A single treatment of 10% w/w L-arabinose significantly reduced glucose peaks (–14%) and insulin peaks (–30%), with delays of 10 and 32 min, respectively. CGM also revealed significant reductions in variability compared with control value: standard deviation (–25%), coefficient of variation (–24%), and mean amplitude of glycemic excursions (–26%). However, no effects on glycemic variability were observed during the controlled diet.

**Conclusions:**

A single treatment of L-arabinose to a sucrose-rich drink reduced insulin and glucose responses in individuals with IFG, but this effect did not extend to a sucrose-rich diet containing complex meals and snacks.

**Trial registration number:**

https://onderzoekmetmensen.nl/nl/trial/27001.

## Introduction

Globally, >90% of diabetes cases are attributed to type 2 diabetes [1]. Effective glycemic control, which involves maintaining blood glucose concentrations within the euglycemic range of 3.9–10.0 mmol/L [2,3], is essential in the progression and management of type 2 diabetes [4]. In a normal physiological state, blood glucose is tightly regulated by homeostatic mechanisms; glucose concentrations fluctuate during the day after food intake but are controlled by the hormones insulin and glucagon, which manage glucose uptake, storage, and release [[4], [5], [6], [7]]. Impaired fasting glucose (IFG) and impaired glucose tolerance (IGT) are generally seen as conditions that progress toward type 2 diabetes, with fasting blood glucose assessments and oral glucose tolerance tests being key diagnostic tests [1]. The prevalence of developing type 2 diabetes in the 5 y after diagnosis is anticipated to be 26% for IGT and 50% for IFG [1,8]. To date, ∼319 million (6.2%) of the world’s adult population have IFG and this is projected to rise to 441 million (6.9%) by the year 2045 [1].

Postprandial blood glucose concentrations are primarily influenced by carbohydrate intake, with different carbohydrate-rich foods inducing varying postprandial glycemic responses [2,9,10]. Some carbohydrates, for example, glucose and sucrose, cause a higher peak response with a sudden decline, whereas others lead to a more gradual and reduced peak response with a gradual decline [7,10]. These responses depend not only on carbohydrate type, but also on the food matrix, gastric emptying rate, and carbohydrate breakdown, glucose absorption, all of which impact glycemic control and variability [6,7,10]. Glycemic variability refers to the fluctuations in blood glucose concentrations, including the magnitude, occurrence, and length of these changes, which are considered a risk factor for the progression of type 2 diabetes [3,11]. Higher postprandial glycemic responses are reflective of insulin resistance and are associated with an increased risk of type 2 diabetes [12]. Lifestyle interventions, particularly dietary modifications, are central to managing glycemic control and reducing glycemic variability [1,4]. The American Diabetes Association recommends limiting added sugars and sugar-sweetened beverages to lower diabetes risk [9].

Recent studies have also investigated functional compounds that inhibit sucrose digestion and glucose absorption to reduce postprandial glycemic responses and improve glycemic control [7,[13], [14], [15]]. L-arabinose is such a compound, which inhibits the enzyme sucrase in the brush border of the small intestine by uncompetitively binding and preventing the hydrolysis of sucrose into glucose and fructose [13]. L-arabinose is a pentose that can be derived through enzymatic hydrolysis from hemicellulose, a polysaccharide abundantly present in the cell walls of plants [[15], [16], [17]]. The exact metabolic pathway of L-arabinose, including whether it serves as an energy source or functions more like a fiber, remains unclear. As L-arabinose has a sweet taste, it can be added to sucrose-rich products without compromising taste [16].

At present, it is not known if L-arabinose supplementation as a functional ingredient in sucrose-rich foods can help individuals with IFG to improve glycemic control. The effects of L-arabinose supplementation on decreasing glycemic and insulinemic responses have been repeatedly demonstrated in healthy individuals [7,[13], [14], [15],^17^,^18^]. Particularly in simple food matrices, such as liquids or jellies, the effects are clear. For example, adding 10% w/w L-arabinose resulted in a reduction of 15% in glycemic and 52% in insulinemic responses in healthy individuals [13]. Similarly, substituting 30% w/w of sucrose in a beverage with L-arabinose resulted in lower glycemic and insulinemic responses of 22% and 67%, respectively [14]. To our knowledge, there are no studies on individuals with IFG or IGT. There is only 1 study that looked at the effects of L-arabinose in individuals with type 2 diabetes; they found lower glucose responses 30 min after the addition of 3% w/w L-arabinose [18].

When looking at foods with more complex food matrices, the data are currently limited to healthy individuals. The available evidence suggests that the effect of L-arabinose is less pronounced as the food matrix becomes more complex [7]; whereas less clear, the effects of L-arabinose were still observed when large quantities of other nutrients such as starch and fat were added to sucrose drinks [13]. However, when added to sucrose-containing cereals [7] or muffins [14], only trends toward lower insulin responses were observed. Considering these results, we expect that individuals with compromised glycemic control, such as IFG, would have an even greater benefit from adding L-arabinose to sucrose-rich meals compared with healthy individuals. Therefore, exploring the potential functionality of L-arabinose in a real-life dietary context with a diet comprising sucrose-rich meals and snacks in individuals with IFG would be valuable.

The current randomized cross-over trial primarily aimed to examine whether a single treatment of 10% w/w L-arabinose added to a sucrose-rich drink lowers glycemic and insulinemic responses in individuals with IFG under acute conditions. Additionally, we explored whether consuming a 15% w/w L-arabinose supplementation before the consumption of real-life sucrose-rich meals and snacks, during a controlled diet, has the potential to reduce glycemic responses as a secondary outcome. We hypothesized that L-arabinose supplementation would reduce the postprandial plasma glucose response compared with no supplementation in both the acute condition and controlled diet.

Continuous glucose monitoring (CGM) was used throughout the trial to monitor the interstitial glucose responses of the individuals with IFG. This novel sensor-based tool has recently emerged to help patients, and their clinical teams, better understand blood glucose fluctuations [11,12,19] and is now also being used for research purposes in nutritional interventions [12,20].

## Methods

### Participants

Males and females with IFG were recruited in Wageningen and surrounding areas, in the Netherlands. For recruitment, emails were sent to individuals through a database of volunteers who had previously expressed their interest in participating in nutritional research studies.

Inclusion and exclusion criteria were assessed through a questionnaire and a fasting blood sample taken during the screening visit. Recruitment was targeted at individuals likely to have poor glycemic control. The inclusion criteria were: age 55–80 y, BMI 25–40 kg/m^2^, no reported weight change >5 kg in the 3 mo before the screening session, IFG defined as 5.6–7.0 mmol/L, or impaired hemoglobin A1c (HbA1c) defined as 39–49 mmol/mol [21]. Exclusion criteria were: diagnosed diabetes, other health-related disorders, use of medication (other than chronic medications) or supplements that could alter the study outcomes, not being able to undertake the measurements according to the study protocol, an alcohol consumption of ≥21 glasses per week for males and 14 glasses per week for females, and a hemoglobin (Hb) level <8.5 mmol/L for males and <7.5 mmol/L for females [22].

An a priori sample size estimation was performed based on the data from a previous study. This study used an identical test drink for the acute test but was performed in healthy individuals [13]. Because individuals with IFG are less likely to have good glycemic control, we expected a similar or slightly larger effect size and standard deviation (SD) in glucose response. Sample size estimations were based on the paired difference between the peak glucose after the intervention drink and the control drink. In the previous study, the glucose peak after the L-arabinose treatment was –1.1 mmol/L lower compared with the control, with an intraperson correlation of 0.2 between measurements [13]. The SD of the effect size (the difference between the glucose peaks after the intervention and control drinks) was 1.1 mmol/L. In addition to using the observed effect size from this previous study, we also estimated the sample size using a clinically relevant mean difference of 1.0 mmol/L in peak glucose and a coefficient of variation (CV) of 15%. This assumption resulted in an estimated sample size of 16 participants. Considering potential dropout, a final sample size of 18 was determined as appropriate for achieving sufficient power to detect differences in peak glucose levels between the intervention and control.

All the procedures were approved by the medical research ethics committee of Wageningen University (ABR no. NL.66.558.081.18) and adhered to the Declaration of Helsinki of 2013. Written informed consent was obtained from all participants and participants received monetary compensation for their contribution. The trial and its primary outcomes were preregistered at the Dutch public trial registry (https://onderzoekmetmensen.nl/nl/trial/27001).

### Experimental design

**FIGURE 1:**
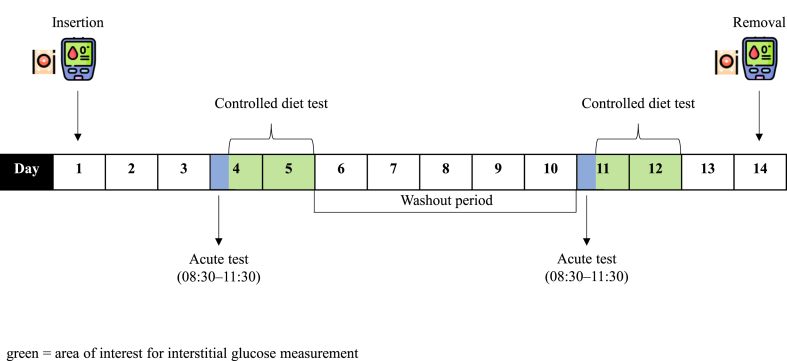
Study timeline, illustrating the day of insertion and removal of the flash glucose monitor and the different outcome measurements performed on certain days within the 14-d study period.

The study was a double-blind randomized cross-over trial with 2 treatments and was carried out between November 2018 and January 2019 at the Division of Human Nutrition and Health, Wageningen University. The intervention consisted of a 2-day test period with 2 tests, an acute test with a sucrose solution followed by a controlled diet containing sucrose-rich meals. To minimize carry-over effects, a washout period of 5 d was used between treatments. Figure 1 shows a schematic overview of the trial.

### Intervention tests

#### Acute test

The control treatment of the acute test was performed with a sucrose-only drink containing 50 g sucrose (Van Gilse). For the intervention treatment, 5 g (10%) L-arabinose (Cosun) was added to the sucrose-only drink. The drinks, after this called *acute test drinks*, were freshly prepared every morning. Sucrose (and L-arabinose) were mixed with 500 mL cold tap water in a Waring blender twice for 10 s (CB102T, Waring). The participants received the following instruction: “Drink the full amount gradually within 5 min with a straw” [13].

#### Controlled diet test

After the acute test, participants received a controlled diet of 10 sucrose-rich meals and snacks to consume within set time periods for 2 d (Table 1). The size of the meals was adjusted for individual differences in energy requirements using the Schofield equation for basal metabolic rate multiplied by a physical activity level of 1.5 [23]. This resulted in 1 participant receiving small-sized meals (estimated energy needs of ∼1800 kcal) and 17 participants receiving medium-sized meals (estimated energy needs of ∼2200 kcal). The medium-sized meals and snacks contained 96.4 g mono- and disaccharides, of which 49.9 g sucrose (10 en%) on the first day, and 106.2 g mono- and disaccharides on the second day of which 51.2 g sucrose (9 en%). As the first test day already included sucrose from the acute test, the total amount of sucrose consumed was 100 g on the first test day (19 en%). See Supplemental Table 1 and Supplemental Figure 1 for more details on the sucrose-rich meals and snacks.

**TABLE 1:** Time frame, snacks, and meals on the 2 test days

Time frame (h)	Day 1	Day 2
08:00–09:00	Acute test drink	Breakfast
11:00–12:00	Lunch	Lunch
14:00–15:00	Snack	Snack
17:00–18:00	Dinner	Dinner
20:00–21:00	Snack	Snack

No breakfast was consumed as part of the controlled diet on the first test day as the acute test was performed.

L-arabinose was provided in a sugar-free lemonade drink, to adjust the dosage to the sucrose content of each meal or snack so that it was always 15% w/w of the sucrose content (Supplemental Table 1). The lemonade drink was freshly prepared on the morning of each test day by dissolving a very low-calorie commercial syrup (Slimpie Framboos syrup) in tap water. The syrup contained a mixture of sweeteners (fruit juice concentrate, cyclamate, acesulfame-K, sucralose) and provided 0.4 g mono- and disaccharides and 13 kcal (56 kJ) per 100 mL. For the control treatment, a lemonade-only drink was prepared in a 1:11 w/w ratio of syrup to water. For the intervention treatment, 25% of the weight of the syrup was replaced by L-arabinose. More details on the syrup can be found in Supplemental Table 2. Participants were instructed to consume a predefined volume of the drink immediately before each sucrose-rich meal or snack.

#### Randomization

Participants and researchers were blinded to the treatments; the randomization and coding were performed by independent researchers. For the randomization, a list with randomized sequences was generated and an independent researcher allocated the participants to either control first or L-arabinose first. A second independent researcher prepared and coded the acute test drinks for the acute test and the lemonade drink for the controlled diet.

### Study procedures

#### Screening

After receiving all the necessary oral and written information regarding the study and signing the study consent form, participants completed the inclusion questionnaire to screen for eligibility. If potentially eligible, they were asked to attend a screening visit including a physical examination. During the screening visit, participants’ height was measured using a stadiometer (SECA) to the nearest 0.1 cm. Body weight was measured using a digital scale (SECA) to the nearest 0.1 kg, and waist and hip circumference were measured with a nonelastic measuring tape (SECA) to the nearest 0.5 cm. Fasting blood samples were taken to assess glucose, HbA1c, and Hb concentrations. Lastly, a trained nurse inspected whether the participants’ veins were suitable for inserting a cannula for frequent blood sampling.

#### Before the test days

The total study lasted for 14 days for each participant, during this whole period (except for the controlled diet) participants were instructed to maintain their habitual diet and activity pattern. Two days before the first 2 test days, a run-in period was initiated after a glucose sensor (see CGM) was placed and from this day onward the consumption of alcohol was prohibited.

On the evening before the test days, participants consumed a low-sugar standardized meal consisting of Nasi Goreng (Albert Heijn) and a small ice cream (Magnum Mini Classic, Unilever). Nasi Goreng is Indonesian fried rice (56% cooked rice, leek, scrambled eggs, 6% ham, and 5% fricandeau). The portion of the meal was 450 g and included 700 kcal per portion (155 kcal/650 kJ, 5.0 g protein, 23 g carbohydrates, 0.8 g fiber, 1.0 g mono- and disaccharides, and 4.5 g fat).

#### On the test days

On the respective test days, the participants visited the Human Research Unit of the university in a fasted state; they were not allowed to drink any form of liquid other than water from 22:00 the previous evening. Participants visited the study center at 07:30 whereafter their study diaries were checked for any deviation from the protocol or adverse events. Their body weight was measured using a digital weighing scale (SECA). A cannula was inserted by a trained nurse into the participants' forearm vein, who remained seated for ≥30 min afterward. An appetite questionnaire, gastrointestinal comfort questionnaire, and a fasted venous blood sample were collected and subsequently, the acute test drink was consumed. Blood samples and appetite questionnaires were collected at 15, 30, 45, 60, 90, 120, and 180 min after the consumption of the acute test drink. After 120 min, a small glass of 135 mL tap water was consumed to prevent thirst/dehydration. Participants were encouraged to remain seated during the whole test session.

After 180 min, the last questionnaires were completed and the last blood sample was drawn, after which the cannula was removed. After the removal of the cannula, the phase with the controlled diet with sucrose-rich meals and snacks started. For this, the first prepared lemonade drink and controlled sucrose-rich meal were given to participants at 12:00 for lunch. After lunch, the participants received packages containing the remaining prepared and preportioned lemonades and sucrose-rich meals and snacks. These needed to be consumed at home for the remainder of the 2-d test period (see Table 1 for the timing of the meals). Participants were told to consume the lemonade drinks before the sucrose-rich meal or snack. On the day after the 2 test days, a researcher phoned the participants to confirm that the participants scanned the glucose sensor as instructed, that the prepared lemonade drink was consumed before eating each meal, and to note if all the provided meals and snacks had been consumed. Participants followed their habitual diet again after the first 2 test days, until the day before the second 2 test days. During the second 2 test days, participants followed the same procedure as for the first 2 test days, only they received the alternate treatment. After the 14-d study period, the glucose sensor was removed.

### Measurements

#### Glucose, insulin, and glucagon

Venous blood samples for glucose analysis were collected in sodium fluoride (NaF) vacutainers and for insulin and glucagon analysis in EDTA vacutainers, which were stored in ice water. The vacutainers were centrifuged for 10 min at 1200×*g* at 4°C, dived over aliquots, put on dry ice, and stored at –80°C until analysis. Plasma glucose concentrations were determined on a Roche cobas c 702-1 analyzer using an enzymatic glucose assay (GLUC3, COBAS, Roche Diagnostics GmbH). Plasma insulin concentrations were measured using a commercial ELISA (catalog no. 10.1113-10, Mercodia Insulin ELISA). Plasma glucagon concentrations were measured using ELISA (catalog no. 10.1271-01, Mercodia Glucagon ELISA). All samples from each participant were analyzed in a single assay.

For all blood parameters, baseline, peak, and time to peak were extracted. The incremental AUC (iAUC_180min_) was calculated by the trapezoidal rule using GraphPad Prism (GraphPad Software).

#### Continuous glucose monitoring

The long-term glycemic effects of L-arabinose supplementation were explored by CGM. A Flash Glucose Monitoring System (Freestyle Libre, Abbott Diabetes Care) was used which measures interstitial glucose every 15 min for a maximum period of 14 d. This sensor does not need calibration with lancets; it is reported to give valid readings ranging from 0.1 to 27.8 mmol/L.

A researcher placed the sensor on the upper arm by embedding the thin, flexible, sterile fiber of 0.5 cm into the skin with a delivery applicator. A skin dressing was placed over the sensor to prevent the removal of the sensor from the skin. Participants were instructed to scan the sensor with the accompanying reader at least once every 8 h. To capture maximal glucose measurements, participants were advised to scan the sensor every time after waking, when they started eating a meal, and just before going to bed.

The glucose sensor captured and stored the glucose readings during the total 14-d study period. For the analyses, only measurements captured during the 2 test days of both treatments were used. Data were obtained from the reader using the Freestyle Libre Software.

#### CGM data processing and glycemic variability

The CGM data were processed and analyzed with RStudio (v2022.07.0 + 548.pro5). More specifically, the shiny application CGMShiny was used to process the data and to calculate and extract measures for glycemic variability. This shiny application is specifically created for CGM data obtained from nutritional research studies [20].

In the shiny application, the default settings for sleep (00:00–06:00) and wake (06:00–00:00) cycles were used [20]; only data during wake cycles were used for analyses. The default settings for the glucose range were used, with 4–10 mmol/L as the healthy range and 3–14 mmol/L as the very low and high thresholds. These ranges correspond to the Freestyle Libre default settings and the international consensus on the use of CGM, 2017 [3]. Furthermore, if readings were missing, a maximum interpolation gap of 45 min was used [24]. Data readings that had larger gaps were reported as missing. There were no missing data points during the acute test; during the controlled diet, the average proportion of missing data points was 2.56% across all participants.

The following parameters of glycemic variability were calculated: mean, SD, CV, and the AUC. The mean amplitude of glycemic excursions (MAGE) was calculated as a parameter for fluctuation. These were chosen based on consensus of use [3,[11], [12],^25^]. Parameters for glycemic variability were extracted separately for the acute test (08:30–11:30) and the controlled diet during the first and second test days (11:40–00:00, day 1; 06:00–00:00, day 2). In addition, fasted readings (taken within 1 or 2 min of the fasted venous blood sampling) were extracted to obtain correlations between measurement methods.

#### Appetite and well-being

The assessment of hunger, degree of satiety, desire to eat, prospective food consumption, and thirst of participants was measured using 100 mm visual analog scales (VAS), anchored by the descriptors “not at all” to “very.” Comfort was also scored on a similar VAS to evaluate overall well-being.

#### Gastrointestinal discomfort

Perceptions of gastrointestinal discomfort, abdominal bloating, regurgitation, flatulence, and nausea were measured through questions with 4 possible answers ranging from “none,” “few,” “moderate,” to “serious” after the last blood sample of each acute test.

#### Blinding and treatment identification

After the last test day, participants completed a questionnaire to assess whether they could taste a difference between treatments (control or intervention) and identify which treatment they had received during the study test days.

### Statistical analysis

Data were analyzed and visualized in RStudio (v2022.07.0 + 548.pro5). Results are reported as mean ± SD and visualized as mean ± SEM unless specified otherwise. For the statistical analysis, *P* values < 0.05 were considered statistically significant and tests were 2-sided. The data were visually inspected for normal distribution by generating histograms and QQ plots of the raw and model residuals.

#### Comparison of treatment effects

The extracted parameters and differences in blood parameters, subjective appetite and well-being, and gastrointestinal discomfort ratings between treatments at different time points were tested independently using a linear mixed-model analysis of variance test. Post hoc tests with Tukey correction were applied using the "lmer" function. Participant number was a random variable, and the fixed factors were treatment (treatment or intervention), time (min), and the interaction between treatment and time. The order of the treatments was not added to the model as testing for an order effect (independent of treatment) did not show an effect. This was tested by running the same linear mixed model with the test days (first or second 2-d test day period) as a fixed variable. Multiple comparisons were adjusted using the Bonferroni method implemented via the p.adjust() function.

#### Correlation analysis

To evaluate the relationship between fasted interstitial glucose and plasma glucose concentrations, a Pearson correlation coefficient (*r*) was calculated including all observations (all time points and treatments).

#### Blinding and treatment identification

The proportion of correct treatment identifications was calculated, and a binomial test was conducted to determine if this proportion significantly deviated from the expected chance level of 50%.

## Results

### Participants

**FIGURE 2:**
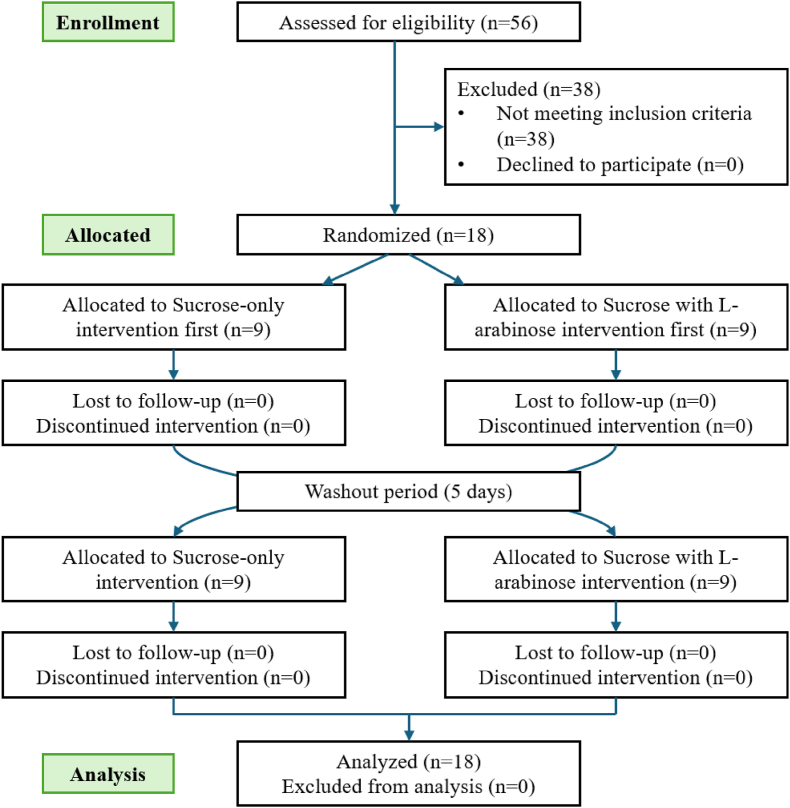
Participant flowchart illustrating the number of participants included and who completed each phase of the randomized cross-over trial and included in the analysis for each intervention.

All 18 participants who were enrolled completed the study and were included in the data analyses. Figure 2 shows a detailed participant flowchart. Participants were predominantly male (78%), with a median age of 75 y. Participants were characterized by a relatively high BMI and waist circumference. On the basis of BMI classifications, 15 participants (83.3%) were categorized as overweight (BMI 25–30 kg/m^2^), and 3 participants were categorized as obese (BMI >30 kg/m^2^). All participants, except 2 males, had a hip–waist ratio higher than 0.90 for males or 0.85 for females. Female participants were generally younger and had slightly lower body weight and waist measurements compared with their male counterparts. Baseline characteristics of the participants can be found in Table 2.

**TABLE 2:** Baseline characteristics of the participants [*n* = 18, mean ± SD, (range)]

	Total (*n* = 18)	Male (*n* = 14)	Female (*n* = 4)
Age (y)	73 ± 5 (58–80)	75 ± 4 (67–80)	68 ± 9 (58–78)
Body weight (kg)	85.4 ± 9.9 (70–106)	86.5 ± 10.8 (70–106)	81.3 ± 4.9 (75–86)
Height (m)	176 ± 6 (165–187)	177 ± 6 (165–187)	172 ± 5 (168–179)
BMI (kg/m^2^)	27.5 ± 2.4 (25–33)	27.5 ± 2.6 (25–33)	27.4 ± 1.1 (26–28)
Waist circumference (cm)	103 ± 8 (90–121)	105 ± 9 (94–121)	99 ± 8 (90–111)
Waist-to-hip ratio	0.96 ± 0.06 (0.87–1.08)	0.98 ± 0.05 (0.87–1.08)	0.91 ± 0.05 (0.88–0.98)

### Acute test

#### Glucose

**FIGURE 3:**
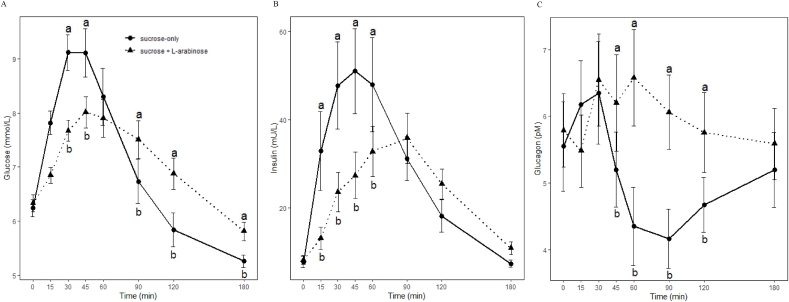
Mean (± SEM) plasma glucose (A), insulin (B), and glucagon (C) responses in participants with impaired fasting glucose (*n* = 18) after the consumption of either a sucrose-only control drink or 10% wt/wt L-arabinose added to the control drink (sucrose + L-arabinose). a,b Indicate significant differences (*P* < 0.05) between the control and intervention treatment at specific time points (mixed-model analysis of variance, post hoc contrasts with Tukey correction).

The postprandial plasma glucose curve exhibited a rapid increase followed by a sudden decline after 45 min after consuming the control drink, sucrose-only. The average peak glucose concentration was 13% (–1.2 mmol/L; F_(1,17)_ = 58.9; *P* < 0.001) lower compared with the control treatment (Table 3). Moreover, statistically significant lower plasma glucose concentrations were observed at 30 and 45 min (Figure 3A). Statistically significant higher plasma glucose concentrations were observed at 90, 120, and 180 min (Figure 3A), suggesting the possibility of a less pronounced decline in plasma glucose concentrations after the addition of L-arabinose.

**TABLE 3:** Baseline and postprandial responses of glucose, insulin, and glucagon after consuming a sucrose-only (control) or sucrose and L-arabinose drink (treatment) during the acute test (*n* = 18, mean ± SD)

	Sucrose only	Sucrose with L-arabinose	Paired mean difference	Confidence interval^1^	*P* value
Glucose					
Baseline (mmol/L)	6.2 ± 0.7	6.3 ± 0.7	0.04 ± 0.03	–0.03, 0.12	0.85
Peak (mmol/L)	9.5 ± 1.5	8.3 ± 1.1	–0.63 ± 0.08	–0.80, –0.46	<0.001
Time to peak (min)	43 ± 13	53 ± 21	5 ± 2	1, 9	0.09
iAUC_180 min_ (mmol/L∗min)	189 ± 98	160 ± 96	–14 ± 8	–31, 2	0.35
Insulin					
Baseline (mU/L)	7.8 ± 5.6	8.2 ± 4.1	0.22 ± 0.33	–0.47, 0.90	1.00
Peak (mU/L)	57.2 ± 41.0	40.0 ± 23.8	–8.61 ± 2.61	–14.1, –3.1	<0.01
Time to peak (min)	46 ± 17	78 ± 30	16 ± 3	10, 21	<0.001
iAUC_180min_ (mU/L∗min)	3712 ± 2878	2835 ± 2024	–438 ± 117	–685, –192	<0.01
Glucagon					
Baseline (pM)	5.6 ± 2.8	5.8 ± 2.3	0.12 ± 0.17	–0.24, 0.48	1.00
Peak (pM)	7.0 ± 2.9	7.5 ± 3.0	0.24 ± 0.19	–0.17, 0.65	1.00
Nadir (pM)	3.5 ± 1.8	4.5 ± 1.9	0.53 ± 0.12	0.28, 0.78	<0.001
Time to peak (min)	59 ± 63	83 ± 66	12 ± 8	–5, 28	0.79
iAUC_180 min_ (pM/L∗min)	68 ± 101	109 ± 106	20 ± 16	–13, 54	1.00

Abbreviation: iAUC, incremental AUC.

1 Upper and lower bounds, 95% confidence interval. Paired mean differences, confidence intervals and *P* values were obtained from post hoc contrasts using the linear mixed model. *P* values were adjusted for multiple comparisons using the Bonferroni method. *P* values were classified as nonsignificant where *P* > 0.05.

#### Insulin

The postprandial plasma insulin curve observed after the consumption of the control drink had a sudden incline and decline in contrast to the addition of L-arabinose where the plasma insulin curve gradually inclined and had an average delay in time to peak of 32 min (Figure 3B, Table 3). The average insulin peak concentration was 30% lower (–17.2 mU/L; F_(1,17)_ = 10.9; *P* < 0.01) and the total insulin response (iAUC) was reduced by 24% when L-arabinose was added to the drink (–877 iAUC_180min,_ mU/L∗min; F_(1,17)_ = 14.1; *P* < 0.01; Table 3). Statistically significant lower plasma insulin concentrations were observed at 15, 30, 45, and 60 min (Figure 3B).

#### Glucagon

The mean plasma glucagon responses were not significantly different after the addition of L-arabinose to the control drink (Table 3). However, statistically significant higher plasma glucagon concentrations were observed at 45, 60, 90, and 120 min with a significantly higher nadir (1.0 pM; F_(1,17)_ = 20.6; *P* < 0.001; Figure 3C).

#### Continuous glucose monitoring

The variability and fluctuation in the interstitial glucose responses were significantly lower after the L-arabinose treatment compared with the control drink (Table 4); the SD was 25% lower (–0.40 mmol/L; F_(1,16)_ = 122.71; *P* < 0.001), the CV 24% (–6.27%; F_(1,16)_ = 121.16; *P* < 0.001) and MAGE 26% (–0.57 mmol/L; F_(1,16)_ = 206.01; *P* < 0.001). The mean interstitial glucose concentration and overall glucose exposure—measured as the AUC—did not differ significantly between the 2 treatment groups (Table 4).

**TABLE 4:** Extracted parameters of the interstitial glucose curves after consuming a sucrose-only (control) or sucrose with L-arabinose drink (treatment) during the acute test (*n* = 18, mean ± SE)

	Sucrose only	Sucrose with L-arabinose	Paired mean difference	Confidence interval^1^	*P* value
Mean (mmol/L)	6.31 ± 0.21	6.28 ± 0.21	–0.02 ± 0.06	–0.14, 0.10	1.00
SD (mmol/L)	1.63 ± 0.09	0.84 ± 0.09	–0.40 ± 0.04	–0.47, –0.32	<0.001
CV (%)	25.8 ± 1.31	13.3 ± 1.31	–6.27 ± 0.57	–7.47, –5.06	<0.001
MAGE (mmol/L)	2.23 ± 0.12	1.10 ± 0.12	–0.57 ± 0.04	–0.65, –0.48	<0.001
AUC (mmol/L∗min)	1166 ± 44	1150 ± 44	–8 ± 15	–40, 24	1.00

Abbreviations: CV, coefficient of variance; MAGE, mean amplitude of glycemic excursions.

1 Upper and lower bounds, 95% confidence interval. Paired mean differences, confidence intervals and *P* values were obtained from post hoc contrasts using the linear mixed model. *P* values were adjusted for multiple comparisons using the Bonferroni method. *P* values were classified as nonsignificant where *P* > 0.05.

The fasted interstitial glucose and plasma glucose concentrations from the acute test had a strong positive correlation, *r*_(34)_ = 0.77, *P* < 0.001, with a 95% confidence interval of (0.58, 0.87).

### Controlled diet test

#### Continuous glucose monitoring

There were no statistically significant differences after the addition of L-arabinose to the control drink for the extracted parameters from the CGM; mean, SD, CV, MAGE, and AUC on the first test day from 11:40 to 00:00 (Table 5). Similarly, the interstitial glucose responses did not differ significantly after the addition of L-arabinose to the control drink for the extracted parameters on the second test day from 06:00 to 00:00 (Table 5).

**TABLE 5:** Extracted parameters of the interstitial glucose curves after consuming a lemonade-only (control) or lemonade with L-arabinose drink (treatment) before each meal during the controlled diet test on 2 consecutive test days (*n* = 18, mean ± SE)

	Lemonade only	Lemonade with L-arabinose	Estimated difference	Confidence interval^1^	*P* value
First test day (11:40–00:00)					
Mean (mmol/L)	6.00 ± 0.15	5.85 ± 0.15	–0.08 ± 0.06	–0.20, 0.05	1.00
SD (mmol/L)	1.21 ± 0.13	1.12 ± 0.13	–0.04 ± 0.04	–0.12, 0.03	1.00
CV (%)	19.7 ± 1.65	18.6 ± 1.65	–0.55 ± 0.56	–1.73, 0.63	1.00
MAGE (mmol/L)	1.78 ± 0.18	1.67 ± 0.18	–0.06 ± 0.06	–0.18, 0.07	0.34
AUC (mmol/L∗min)	4230 ± 112	4076 ± 112	–77 ± 40	–161, 7	1.00
Second test day **(0**6:00–00:00**)**					
Mean (mmol/L)	6.32 ± 0.18	6.18 ± 0.18	–0.07 ± 0.05	–0.17, 0.04	0.91
SD (mmol/L)	1.38 ± 0.13	1.35 ± 0.13	–0.01 ± 0.03	–0.08, 0.05	1.00
CV (%)	21.6 ± 1.48	21.3 ± 1.48	–0.13 ± 0.4	–1.02, 0.77	1.00
MAGE (mmol/L)	1.99 ± 0.17	1.95 ± 0.17	–0.02 ± 0.05	–0.12, 0.09	0.77
AUC (mmol/L∗min)	6641 ± 196	6428 ± 196	–107 ± 71	–257, 44	1.00

Abbreviations: CV, coefficient of variance; MAGE, mean amplitude of glycemic excursions.

1 Upper and lower bounds, 95% confidence interval. Paired mean differences, confidence intervals and *P* values were obtained from post hoc contrasts using the linear mixed model. *P* values were adjusted for multiple comparisons using the Bonferroni method. *P* values were classified as nonsignificant where *P* > 0.05.

#### Subjective appetite and comfort

All measures of subjective appetite changed during the acute test (F_(15, 239.01)_ = 6.86, *P* < 0.001). Participants’ level of comfort did not change during the acute test (F_(15, 239)_ = 0.96, *P* = 0.50). No significant treatment × time effect was seen for subjective appetite and comfort (F_(15,152.56)_ = 1.38, *P* = 0.16).

#### Gastrointestinal discomfort

Overall, no reports of serious gastrointestinal discomfort were made. The measures of gastrointestinal discomfort did not significantly change during the controlled test days and no significant differences were seen between the control and intervention treatment (F_(3, 64)_ = 1.27, *P* = 0.29).

#### Blinding and treatment identification

Answers to the debriefing questionnaire showed that in 12 of 36 (33%) instances, participants were correct in identifying which treatment they received (*P* = 0.07), with a 95% confidence interval of (0.19, 0.51). Note that study participants were not informed that the study had a cross-over design.

## Discussion

Supplementing sucrose-rich foods with L-arabinose may help reduce blood glucose responses in individuals with IFG, who are at risk of developing type 2 diabetes. Our study showed that adding L-arabinose to a simple sucrose solution (10% w/w) significantly reduced and delayed peaks in blood glucose concentrations. Moreover, CGM-based parameters showed that the addition of L-arabinose decreased glucose variation and fluctuation. Furthermore, we observed clear effects on insulin, resulting in a lower and delayed insulin peak and reduced overall response. However, consuming 15% w/w L-arabinose preceding various sucrose-rich meals and snacks—mimicking a real-life high-sucrose diet—did not affect CGM-based parameters of glycemic variability.

To our knowledge, this study was the first to show the effects of L-arabinose on glucose homeostasis in individuals at risk of developing type 2 diabetes. Our participant group consisted of an older population of mostly males with central obesity and was screened for having subclinical IFG by fasting glucose levels or elevated HbA1c. Because baseline levels can vary significantly across days, we evaluated fasting glucose levels during the study to confirm IFG levels; all but 1 participant had fasting baseline levels between 5.6 and 7.0 mmol/L. Excluding this participant from the analyses did not change the conclusions of our study, confirming the validity of the conclusions for individuals with IFG.

The acute test showed effects of L-arabinose consistent with the findings in healthy individuals. After L-arabinose supplementation to the sucrose-rich drink, we observed an average decrease of 1.3 mmol/L (14%) in peak glucose levels and the average peak in insulin levels was substantially lower (–17 IU/mL, 30%). The absolute declines in peak concentrations were similar to those observed in our previous study with healthy individuals [13], which reported decreases of 1.1 mmol/L and 20 mU/L in glucose and insulin, respectively. Proportionally, the effects were somewhat smaller in our current study, likely due to the elevated fasting glucose and insulin levels in our participant group.

In addition to the effects on glucose and insulin peaks, we noted significant changes in the timing of the peaks, which were also seen in healthy individuals [13]. The time to peak of insulin was delayed by ∼30 min. This delay was more pronounced compared with earlier studies involving healthy individuals [13,15]. In contrast, the study by Inoue involving individuals with type 2 diabetes did not report any delays in peak times [18]. However, this study included only 10 participants and used a relatively low dosage of 3% w/w.

The delay in insulin peak after L-arabinose treatment may be attributed to slower gastric emptying and a glucagon-like peptide-1 (GLP-1) response due to the presence of undigested sucrose in the small intestine [13], resulting from the binding of L-arabinose to sucrase [4,14]. GLP-1 is known to delay gastric emptying and initiate the “ileal break” [26,27]. We observed an increase in GLP-1 responses in our previous study [13], although we did not measure GLP-1 levels in this study.

Furthermore, there seems to be a clear relationship between the insulin and glucagon response during the acute test. Although the insulin response to sucrose resulted in a higher peak compared with L-arabinose, a corresponding decrease in glucagon levels was observed, reaching a significant nadir in the presence of sucrose-only. This effect was attenuated with the addition of L-arabinose, which may slightly diminish the positive effects on glycemic and insulinemic responses. With glucagon levels remaining elevated with the addition of L-arabinose, there may be continued stimulation of endogenous glucose release, potentially moderating the overall metabolic response.

The controlled diet test with sucrose-rich meals and snacks was an exploratory part of the current study. We gave our participants 15% w/w L-arabinose, which is relatively high. However, we can only speculate why we did not see any effects of L-arabinose supplementation on the CGM-based parameters. In previous studies, we also observed smaller effects when foods contained other nutrients and had more complex matrices compared with simple liquids [7,14]. We may speculate that our population had delayed gastric emptying. Therefore, the effects of L-arabinose may have been overshadowed by the glucose already entering the small intestine at a slower rate. Literature indicates that patients with diabetes and older populations have slower gastric emptying [28,29], resulting in lower and later glucose peaks [28]. Although we used a higher dosage than the acute test and the previous studies we performed, a dose higher than 15%w/w may be needed to see any effects in a real-life context in individuals with slower gastric emptying.

We may have missed effects because we used CGM. Although the use of CGM allowed long-term monitoring of glucose levels in a home setting, these sensors measure interstitial glucose concentrations, which may be less sensitive to fluctuations compared with venous blood. Additionally, the sensors do not give any information on insulin levels or other blood markers that may provide more mechanistic insights. Because our participants also wore the sensor during the acute test, we had the opportunity to validate the sensor readings with results from the venipuncture. We found a strong correlation in fasting results (*r* = 0.77).

Given that our participant group was predominantly male with central obesity, this could influence the generalizability of our findings to other groups at risk of developing type 2 diabetes. It is also expected that the effects of L-arabinose might be greater in individuals with IGT. Additionally, other functional effects of L-arabinose on metabolism remain to be explored. It is plausible that L-arabinose may also impact other metabolic markers or hormones beyond glucose and insulin, warranting further investigation.

To conclude, adding 10% w/w L-arabinose to sucrose-rich drinks reduces both insulin and glucose responses in individuals with IFG in the absence of any additional simultaneous consumption. In addition, CGM-based parameters of glucose variability and fluctuation decreased. Therefore, L-arabinose could serve as a functional ingredient for supplementation or partial sucrose substitution without the loss of sweetness. However, how L-arabinose can be integrated and the extent of its glycemic lowering effects in more complex daily meals or its clinical significance remains a topic to be further investigated. Future research should focus on the real-life implementation of L-arabinose, utilizing repeated intravenous measurements of glucose, insulin, and other biomarkers of glucose homeostasis. Lastly, as there is an increasing interest in biomonitoring using CGM and other biosensors, the validation of these sensors has become critical. Continued development and optimization of data software are necessary to process and analyze CGM data from nutritional studies and communicate results easily interpretable by researchers and clinicians.

## Author contributions

The authors’ responsibilities were as follows – KP, MM, CP: designed the research; KP: conducted the research; LP, KP: analyzed the data and wrote the manuscript; LP, MM, KMA, JJ, CP: revised and edited the manuscript; and all authors: read and approved the final manuscript.

## Data availability

Data described in the manuscript, code book, and analytic code can be obtained upon reasonable request from the corresponding author.

## Funding

This work was supported by the Pulp2Value project from the BioBased Industries Joint Undertaking under the European Union’s Horizon 2020 Research and Innovation Programme (under grant agreement No 669105), and part of a studentship undertaken by Leoné Pretorius funded by Bournemouth University and Wageningen University and Research.

## Conflict of interest

MM reports equipment, drugs, or supplies was provided by Cosun Innovation Center. KP reports equipment, drugs, or supplies was provided by Cosun Innovation Center. MM reports financial support was provided by European Commission. KP reports financial support was provided by European Commission. KMA reports a relationship with Unilever Innovation Centre Wageningen BV that includes: funding grants. KMA reports a relationship with ILSI North America that includes: funding grants and speaking and lecture fees. KMA reports a relationship with International Sweeteners Association that includes: funding grants and speaking and lecture fees. KMA reports a relationship with The Coca Cola Company that includes: funding grants and speaking and lecture fees. KMA reports a relationship with EatWell Global that includes: speaking and lecture fees. If there are other authors, they declare that they have no known competing financial interests or personal relationships that could have appeared to influence the work reported in this article.
