# Supplementary material for: Effects of L-Arabinose on Glycemic Responses After the Consumption of Sucrose-Rich Foods in Individuals with Impaired Fasting Glucose: A Randomized Controlled Cross-Over Trial
Source: J Nutr. 2025 Jul 1;155(9):3030–9. doi: 10.1016/j.tjnut.2025.06.028 (PMC12799425; doi:10.1016/j.tjnut.2025.06.028)
Supplement: multimedia component 1 [file mmc1.docx]

**SUPPLEMENTARY MATERIALS**

**SUPPLEMENTARY TABLE 1**

The volume of the lemonade drinks and meal constituents consumed by participants during the controlled diet.

|  | **L-arabinose**  **(g)** | **Lemonade Volume**  **(g)** | **Meal constituents** | **Total Mono- and Disaccharides (g)** |
| --- | --- | --- | --- | --- |
| **First test day** | | | | |
| Acute test | 5.0 | 505 (no syrup) | Water, sucrose | - |
| Lunch | 3.0 | 144 | Brown wheat bread, low-fat margarine, Gouda cheese 48+, jam, fruit-flavored colored sprinkles, shoulder ham, cherry tomatoes, peppermint | 19.8 |
| Snack | 4.0 | 192 | Cake, pear, boiled sweets, mandarin | 26.5 |
| Evening meal | 4.2 | 202 | Curly kale with mashed potatoes and smoked sausage, vanilla custard | 28.3 |
| Snack | 3.3 | 158 | Mandarin, Bastogne shortbread biscuit, apple | 21.8 |
| **Second test day** | | | | |
| Breakfast | 2.4 | 155 | Brown wheat bread, low-fat margarine, Gouda cheese 48+, fruit-flavored colored confetti, jam, crispbread | 16.1 |
| Lunch | 2.2 | 106 | Brown wheat bread, low-fat margarine, Gouda cheese 48+, jam, fried minced meatloaf, raw carrots | 14.5 |
| Snack | 3.5 | 168 | Jodekoek (Dutch biscuit), orange, boiled sweets | 23 |
| Evening meal | 4.2 | 202 | Nasi, atjar tjampoer, prawn crackers, chocolate custard | 28.5 |
| Snack | 3.3 | 158 | Banana, apple, spiced biscuits | 24.1 |
| **Total** | 30.1 | 1445 |  | 202.6 |

**SUPPLEMENTARY TABLE 2**

Nutrient information and ingredients of the Slimpie framboos syrup

| **Nutrient information** | **Per 100 ml** |
| --- | --- |
| Energy (kJ) | 56 |
| Energy (kcal) | 13 |
| Fat (g) | 0 |
| Carbohydrates (g) | 4.1 |
| - - Of which sugars (mono - & disaccharides) | 0.4 |
| Fiber (g) | 0 |
| Protein (g) | 0 |
| Salt (g) | 0.33 |
| **Ingredients**: water, citric acid, 2% fruit juice from concentrate (raspberry juice 1.1%, apple juice), aroma, elderberry juice, cyclamate, acesulfame-K, sucralose, E211, E202, E331, E150d. | |

**
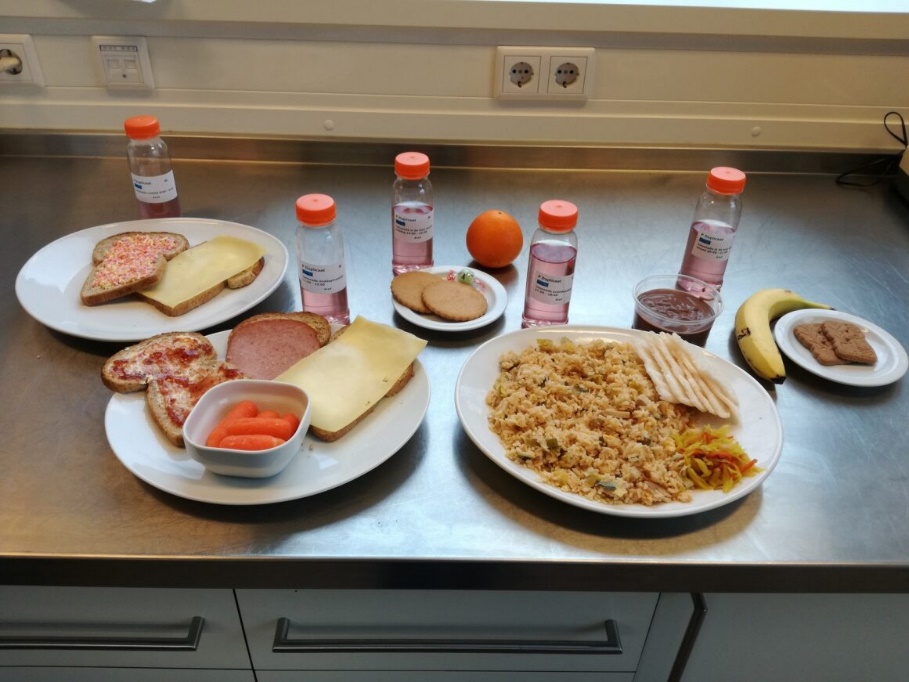

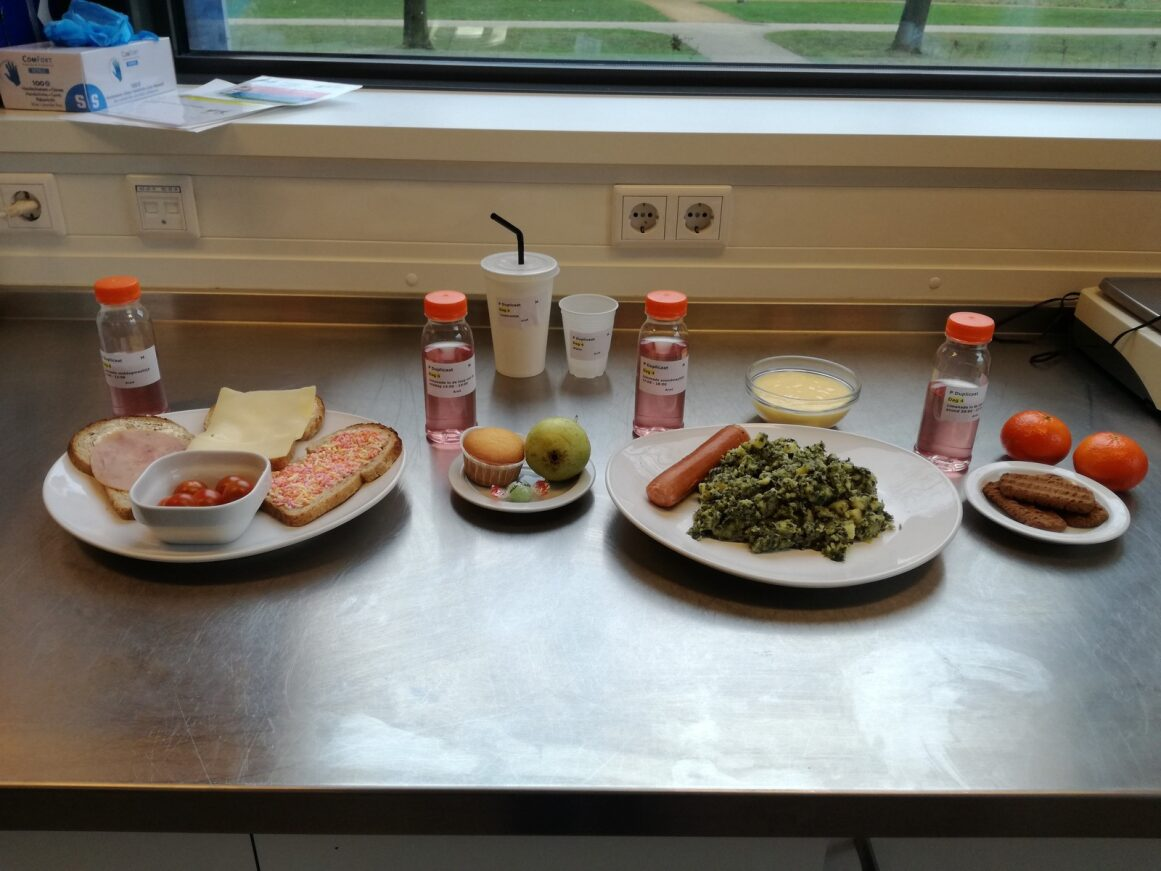

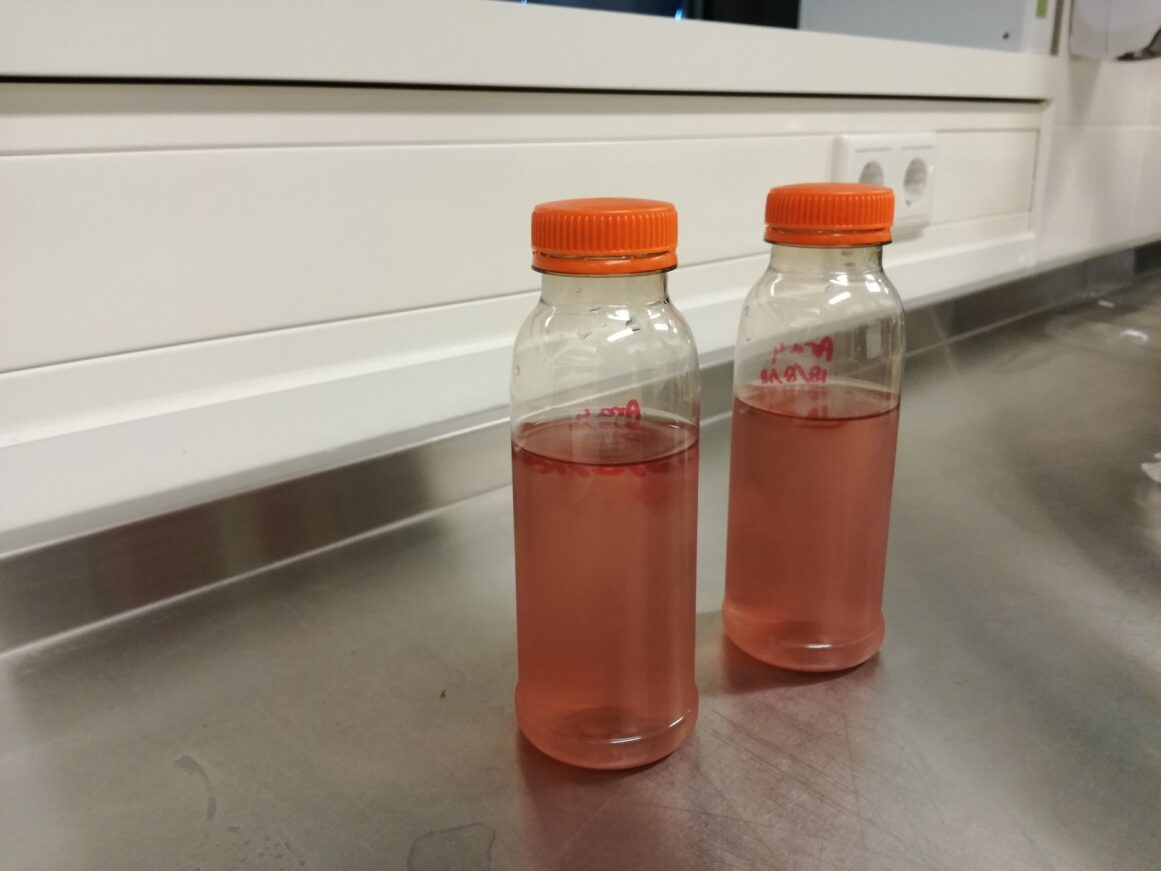
SUPPLEMENTARY FIGURE 1**

**Supplementary Figure 1:** Example of the lemonade drink (enlarged in A) consumed before consuming the different types of sucrose-rich meals and snacks during the controlled diet test on the first and second test days (B, C).

Second test day

First test day

C

B

A
